# Pretreatment with serine protease inhibitors impairs *Leishmania amazonensis* survival on macrophages

**DOI:** 10.1186/s13071-024-06630-w

**Published:** 2025-01-23

**Authors:** Patrícia de Almeida Machado, Pollyanna Stephanie Gomes, Elaine Soares Coimbra, Herbert Leonel de Matos Guedes

**Affiliations:** 1 Laboratório de Imunologia Clínica, Instituto Oswaldo Cruz, Fundação Oswaldo Cruz-Fiocruz, Rio de Janeiro, RJ 21040-360 Brazil; 2 Laboratório de Imunobiotecnologia, Instituto de Microbiologia Paulo de Góes, Universidade Federal Do Rio de Janeiro, Rio de Janeiro, RJ 21941-902 Brazil; 3 Núcleo de Pesquisas Em Parasitologia (NUPEP), Instituto de Ciências Biológicas, Universidade Federal de Juiz de Fora, Juiz de Fora, MG 36036-900 Brazil; 4 Laboratório de Imunofarmacologia, Instituto de Biofísica Carlos Chagas Filho (IBCCF), Universidade Federal Do Rio de Janeiro, Rio de Janeiro, RJ 21941-902 Brazil

**Keywords:** Serine proteases, Serine proteases inhibitors, *Leishmania amazonensis*

## Abstract

**Background:**

Leishmaniases are neglected tropical diseases with great clinical and epidemiological importance. The current chemotherapy available for the treatment of leishmaniasis presents several problems, such as adverse effects, toxicity, long treatment time, and parasite resistance. The discovery of new therapeutic alternatives is extremely essential, and the discovery of cellular targets is a tool that helps in the development of new drugs. Serine proteases emerge as important virulence factors in the *Leishmania* genus, as they participate in important processes involved in their infectivity, virulence, and survival. In this work, we evaluated the leishmanicidal effect of different serine protease inhibitors (Benzamidine, PF-429242, PMSF, TLCK, and TPCK). Additionally, we determined the implication of pretreatment with these inhibitors on the entry and survival of parasites within macrophages, as well as the conversion of promastigotes into amastigotes, to discover the importance of serine proteases in the establishment of infection and, consequently, as targets for new drugs for *Leishmania*.

**Results:**

In general, the inhibitors had low toxicity in host macrophages, and three showed some effect in promastigote and amastigote forms of *L. amazonensis* (PF-429242, TLCK, and TPCK). Using a short incubation interval, we pretreated *L. amazonensis* promastigotes with these five compounds before in vitro infection. Pretreatment with PF-429242, TLCK, and TPCK considerably compromised the survival of these parasites inside host macrophages, without altering the entry of promastigotes into these cells and differentiation into amastigotes. In addition, treatment with PF-429242 and TPCK was able to reduce the serine proteases’ enzymatic activity using subtilisin substrate on *L. amazonensis* promastigote lysate.

**Conclusions:**

This work highlights the importance of serine proteases in *L. amazonensis* as a possible target for new therapeutic alternatives in *Leishmania* spp.

**Graphical Abstract:**

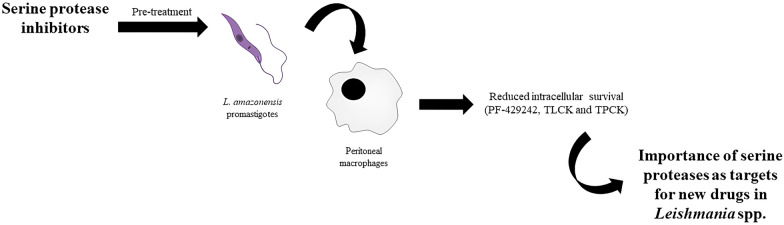

**Supplementary Information:**

The online version contains supplementary material available at 10.1186/s13071-024-06630-w.

## Background

Leishmaniases are neglected tropical diseases [1] caused by parasites from the *Leishmania* genus and transmitted to humans by the bite of infected female phlebotomine sandflies [2]. Depending on the *Leishmania* species, it can manifest as cutaneous leishmaniasis (CL) and visceral leishmaniasis (VL) [3]. CL is the most common form, and VL is the most severe form, which can lead to death if not treated. More than 1 billion people live in endemic areas at risk of infection. It is estimated that more than 1 million new cases of CL and 30,000 new cases of VL occur annually [2].

First-line drugs to treat leishmaniasis are pentavalent antimonials, such as *N*-methyl glucamine antimoniate, which is available in Brazil. This drug is very toxic and can cause cardiac and hepatic dysfunction. In addition, long-term treatment and parenteral administration impair adherence to treatment. As second-line treatments, amphotericin B and its liposomal formulations, pentamidine, paromomycin, and miltefosine, have variable antileishmanial efficacy, toxicity, adverse effects, long-term treatment, parenteral administration, and high cost. Also, cases of parasite resistance for all available leishmanicidal drugs have been seen frequently, depending on the species and geographic region [1]. On the basis of this, there is an urgent need for cheaper, safer, and more effective antileishmanial drugs [4]. The discovery of new therapeutic targets in *Leishmania* spp. is essential for the discovery of new therapeutic alternatives.

Proteases are involved in many physiological and pathological processes. These proteins are considered a good target for drug development, because their structure and enzymatic mechanisms are well known [5, 6]. Therapies based on proteases are already used in clinical medicine, with the use of HIV protease inhibitors for the treatment of AIDS being a great example [6, 7]. Serine proteases constitute one of the major groups of proteases [8]. In *Leishmania* spp., serine proteases are involved in the invasion and proliferation of the parasite in the host cell [9], differentiation of promastigotes in amastigotes, and resistence to oxidative stress [10]. It is also interesting to highlight that the construction of knockouts for subtilisin did not give rise to viable parasites (such as *L. major*) or it gave rise to parasites with no capacity to differentiate to amastigotes (such as *L. donovani*) [10, 11]. These facts demonstrate that serine proteases have great importance for *Leishmania* parasites, making them cellular targets for different compounds.

In this line, this work evaluated the leishmanicidal effect of different serine protease inhibitors already known in the literature and determined the implication of pretreatment with these inhibitors, using a nonlethal dose, on the infection and survival of *Leishmania amazonensis* in host cells, to enable an evaluation of the participation of serine proteases, especially subtilsin, in this process.

## Methods

### Compounds

Serine protease inhibitors already known, such as benzamidine, PF-429242, PMSF, TLCK, and TPCK, were purchased from Sigma-Aldrich (St. Louis, MO, USA) and dissolved in deionized water or DMSO (Sigma-Aldrich).

### Parasites

Two strains of *Leishmania amazonensis* were used in this work: IFLA/BR/1967/PH8 (Wild-type—WT) and IFLA/BR/1967/PH8 transfected with the gene for red fluorescent protein (RFP). *L. amazonensis* WT was cultivated in Brain Heart Infusion (BHI) medium (Kasvi, São José dos Pinhais, PR, Brazil) supplemented with 10% of fetal bovine serum (FBS) (Cultilab, Campinas, SP, Brazil), 0.1% penicillin and streptomycin solution (Sigma-Aldrich), 5 mg/mL hemin (Sigma-Aldrich), and 0.5 mg/mL folic acid (Sigma-Aldrich). *L. amazonensis* RFP was grown in 199 medium (HiMedia Laboratories Pvt. Ltd., Mumbai, India) supplemented with 10% FBS, 0.1% penicillin and streptomycin solution, 5 mg/mL hemin, 0.5 mg/mL folic acid, 0.2 mg/mL D-biotin (Sigma-Aldrich), 4 mg/mL adenine (Sigma-Aldrich), and MEM vitamin solution (Thermo Fisher Sci., Waltham, MA, USA).

### Mice

Six- to eight-week-old female BALB/c mice were obtained from the Central Animal Facility of the Universidade Federal do Rio de Janeiro (UFRJ). All procedures for the use of animals were performed according to protocol approved by the Ethical Committee for Animal Handling (CEUA 080/2018).

### Antipromastigote assay

*Leishmania amazonensis*–WT promastigotes, in the logarithmic phase of growth, were transferred to 96-well culture plates (final concentration 2 × 10^6^ cells/mL) and exposed to different concentrations of inhbitors during 72 h at 25 °C. The cell viability was evaluated by the 3-(4,5-dimethylthiazol-2-yl)-2,5 diphenyltetrazolium bromide (MTT) method (obtained from Sigma-Aldrich). The inhibitory concentration of 50% of parasite growth (IC_50_) values were calculated using GraphPad Prism 8 software (Erithacus Software Ltd). Three independent experiments in triplicate were performed. Amphotericin B (Cristália, São Paulo, SP, Brazil) was used as a reference drug.

### Antiamastigote assay

Peritoneal macrophages from BALB/c mice were obtained by peritoneal lavage and distributed in 24-well plates at 2 × 10^6^ cells/mL in RPMI medium (Cultilab) containing 10% FBS and a 0.5% penicillin and streptomycin solution (complete RPMI). The plates were incubated for 1 h at 37 °C and 5% CO_2_, washed with phosphate-buffered saline (PBS), and then reincubated in complete RPMI medium overnight. The cells were washed again with PBS and infected with stationary growth phase *L. amazonensis* (RFP) promastigotes at a 10:1 ratio for 4 h at 33 °C. The plates were washed with PBS and the inhibitors were added at different concentrations and incubated for 72 h. After this time, each well was scraped to remove attached cells and the reading was taken with a spectrofluorimeter (FLx800, BioTek Instruments, Winooski, VT, USA) at 530 nm and 590 nm of excitation and emission, respectively. Some wells did not receive treatment (control). All assays were performed in triplicate and repeated three times. Results were calculated as the percentage of amastigote survival compared with the nontreated control group, and IC_50_ values were determined using GraphPad Prism 8 software.

### Cytotoxicity assay

To determinate the toxicity of the inhibitors on mammalian cells, peritoneal macrophages from BALB/c mice, obtained by peritoneal lavage, in concentration of 2 × 10^6^ macrophages/mL, were adhered in 96-well culture plates overnight at 37 °C with 5% CO_2_. The inhibitors were then added in different concentrations and incubated for 72 h at 37 °C and 5% CO_2_. Cell viability was also assessed by the MTT method. Cytotoxic concentration of 50% of macrophages (CC_50_) values were calculated using GraPadPrism 8 software. Three independent experiments in triplicate were performed.

### Viability of promastigotes after 1 h of treatment with inhibitors

*Leishmania amazonensis*-RFP promastigotes were treated with different concentrations of inhibitors during 1 h at 25 °C and were washed with PBS. Then, these cells had their viability determined by fluorescence analysis using a spectrofluorimeter (time = 0 h) and were also reincubated in complete RPMI medium for subsequent analysis of cell viability after 24 h, 48 h, and 72 h (times = 24 h, 48 h, and 72 h) by fluorescence analysis.

### Promastigote treatment and infection

*Leishmania amazonensis*-RFP promastigotes were pretreated with different concentrations of inhbitors during 1 h at 25 °C. The cells were washed with PBS and used to infect peritoneal macrophages from BALB/c mice (10:1 ratio), not pretreated or pretreated with 0.5 ng/mL IFN-γ overnight, in 24-well plates. After 4 h of interaction, the plates were washed with PBS and (1) scraped to remove attached cells and read using a spectrofluorimeter (time = 0 h) or (2) incubated at 33 °C with 5% CO_2_ for 24 h, 48 h, and 72 h for later scraping and reading (times = 24 h, 48 h, and 72 h). Three experiments were performed, each one with three replicates.

The same experiment was repeated, but using 13 mm glass coverslips in 24-well plates. The fixation with ethanol and staining with Giemsa were performed. Subsequently, the slides were examined in an optical microscope (Olympus BX53, Shinjuku, Tokyo, Japan) and photographed (Olympus DP73, Shinjuku, Tokyo, Japan).

### Determination of nitric oxide (NO) levels

Supernatant from cultures of *L. amazonensis*-infected macrophages, in which promastigotes were pretreated or not with with PF-429242 or TPCK, was collected, and NO levels were determined by the Griess method [12]. NO levels were determined in aliquots of 50 µL of the supernatant, to which 50 µL of the Griess reagent were added (Griess Reagent: 1% sulfanilamide in 2.5% of H_3_PO_4_ + 0.1% N-1-diidrocloride naftiletilenodiamine in 2.5% H_3_PO_4_), in a 96-well microplate. Nitrite content was quantified by comparison with a sodium nitrite standard curve. The absorbance was measured at 540 nm using a spectrophotometer (Multiskan EX, Thermo Electron Corporation, Vantaa, Finland). The assays were carried out in duplicate and three independent experiments were realized. Macrophages prestimulated with 0.5 ng/mL IFN-γ overnight and infected with *L. amazonensis* promastigotes without pretreatment were used as positive controls.

### Enzymatic assay

The enzymatic assay was conducted in a final volume of 200 µL per well, containing 1 µM of substrate, supernatant of lysate of 3.7 × 10^6^ parasites, 2.5 µM of inhibitors (PF-429242 and TPCK) or 3 µM of E-64 in Tris-buffered pH 8. Substrate ABZ-V-F-R-S-L-K-Q EDDnp was used, which was provided by Dr. Luiz Juliano Neto from the Department of Biophysics of UNIFESP-EPM and is described in Alves, L.C. et al. [13]. The lysate was prepared with promastigotes in stationary phase of growth, washed three times with PBS pH 7.2, and in the last wash it was resuspended in Tris-buffered pH 8 with 1% NP40. The parasites were submitted to five rapid freeze–thaw cycles in liquid nitrogen. The lysate was centrifuged at 20,000*g* for 10 min at −4 °C. The supernatant was stored at −20 °C until use. The read was carried out using the spectrofluorimeter SoftMax M3 with excitation/emission of 320/420 nm.

### Statistical analyses

Statistical analyses were performed by using GraphPad Prism 8 software. Statistical differences between mean values were evaluated by applying one-way ANOVA with Tukey’s or Dunnett’s post-test. The IC_50_ and CC_50_ were obtained by nonlinear regressions also using GraphPad Prism 8.

## Results

### Effect of inhibitors on macrophages and* L. amazonensis*

We performed antipromastigote, antiamastigote, and citotoxicity assays to determine the IC_50_ and CC_50_ values for different serine protease inhibitors in *L. amazonensis* and peritoneal macrophages. Table 1 shows that serine protease inhibitors have low toxicity against mammalian cells after 72 h of incubation, with all CC_50_ values above 100 µM. The most toxic inhibitors were TLCK (CC_50_ = 103.6 µM) and TPCK (CC_50_ = 138.8 µM). Additionally, Table 1 shows that, after 72 h of incubation, of the five serine protease inhibitors, three were effective in promastigote and amastigote forms of *L. amazonensis* (PF-429242, TLCK and TPCK), with PF-429242 being the most effective in promoastigotes (IC_50_ = 0.83 µM) and TPCK the most effective in amastigotes (IC_50_ = 14.2 µM). In addition, after 72 h of incubation, benzamidine and PMSF were not effective up to the maximum concentration tested, which was 200 µM (Table 1).

**Table 1:** Effect of serine protease inhibitors in peritoneal macrophages and *Leishmania amazonensis* promastigotes and amastigotes

Compound	Class	Peritoneal macrophages CC_50_^a^	Antileishmanial effect in *Leishmania amazonensis* IC_50_^b^		Selectivity index (SI)^c^	
			Promastigote	Amastigote	Promastigote	Amastigote
Benzamidine	Serine protease inhibitor	239.8 (191.7–299.9)	> 200.00	> 200.00	–	–
PF-429242	Serine protease inhibitor−subtilisin inhibitor	189.07 (176.78–201.36)*	0.83 (0.71–0.95)*	119.68 (101.61–137.75)*	227.8	1.6
PMSF	Serine protease inhibitor	269.1 (212.5–340.8)	> 200.00	> 200.00	–	–
TLCK	Serine protease inhibitor	103.6 (77.82–137.9)	52.54 (34.07–81.04)	139.80 (137.80–141.9)	2.0	0.7
TPCK	Serine protease inhibitor	138.8 (128.4–150.1)*	14.6 (10.9–19.6)*	14.2 (7.7–26.2)*	9.5	9.8
AMB	—	21.78 (18.75–37.5)	0.12 (0.11–0.13)	0.18 (0.12–0.26)		

^a^Cytotoxic concentration of 50% of macrophages (CC_50_). ^b^Inhibitory concentration of 50% of parasite growth (IC_50_). CC_50_ and CI_50_ were calculated after 72 h of incubation. ^c^Selectivity index: CC_50_ in macrophages/IC_50_ in parasite.

*Previously published data

**Fig. 1:**
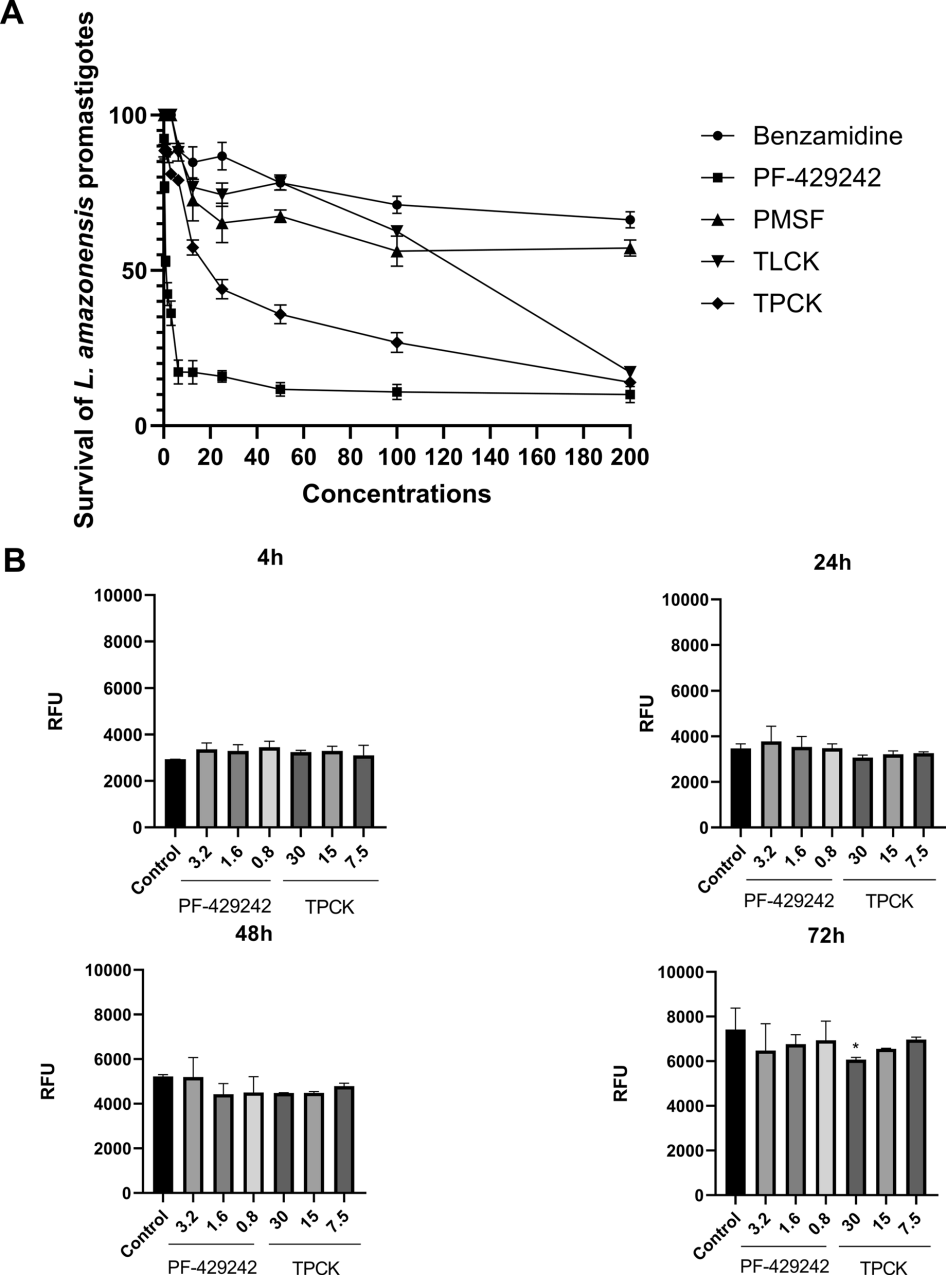
Effect of 1 h and 72 h of treatment with serine protease inhibitors against *L. amazonensis* promastigotes. **A** *L. amazonensis* promastigotes were incubated with the compounds for 72 h and cell viability was determined by the MTT method. Survival data were calculated on the basis of the negative control. The graph was constructed using the GraphPad Prism program. **B*** L. amazonensis* promastigotes were incubated with PF-429242 or TPCK for 1 h, washed with PBS, and placed back in culture medium containing 10% SBF. Cell viability was then determined immediately after washing (0 h) and after 24 h, 48 h, and 72 h by MTT method. Graphs were constructed using the GraphPad Prism program. Statistical analyses were performed by a one-way ANOVA followed by Tukey’s post-test to compare the untreated control group with the other treatments: *p < 0.05

Figure 1A shows the survival curves of *L. amazonensis* promastigotes after 72 h of treatment with inhibitors. As already shown above, the most effective compounds against promastigote forms were firstly PF-429242, followed by TPCK.

In parallel, after 1 h of treatment with the inhibitors, the promastigotes were washed with PBS and placed back in culture medium containing 10% FBS. Cell viability was then determined immediately after washing (0 h) and after 24 h, 48 h, and 72 h. Treatment of promastigotes for 1 h with benzamidine, PMSF, and TLCK did not change the viability of the parasites, when compared with the control, at 0 h or after 24 h, 48 h, and 72 h of incubation (data not shown). Figure 1B shows that, in general, 1 h of treatment with the inhibitors PF-429242 and TPCK was also not able to change cell viability, when compared with the negative control, as the fluorescence intensity in the control and treated cells were similar. Only treatment with 30 µM TPCK caused a slight reduction in the viability of *L. amazonensis* promastigotes after 72 h of incubation (Fig. 1B).

### Pretreatment with inhibitors affects the survival of* L. amazonensis* within macrophages

To evaluate the importance of serine proteases in *L. amazonensis*, we used these inhibitors to pretreat *L. amazonensis* promastigotes and then infected peritoneal macrophages with untreated promastigotes (negative control) or pretreated with the different inhibitors for 1 h. For benzamidine and PMSF, which are not effective against promastigotes, we chose a high concentration (200 µM) and a low concentration (6.25 µM). For the compounds that showed an effect on promastigote forms, we used the following: for TLCK, 2× the IC_50_ value (100 µM) and a concentration that inhibited around 10% of the parasite’s viability (6.25 µM); for PF-429242, concentrations 1×, 2×, and 4× the IC_50_ value (0.8 µM, 1.6 µM, and 3.2 µM); and for TPCK, concentrations 0.5×, 1×, and 2× the IC_50_ value (7.5 µM, 15 µM, and 30 µM).

**Fig. 2:**
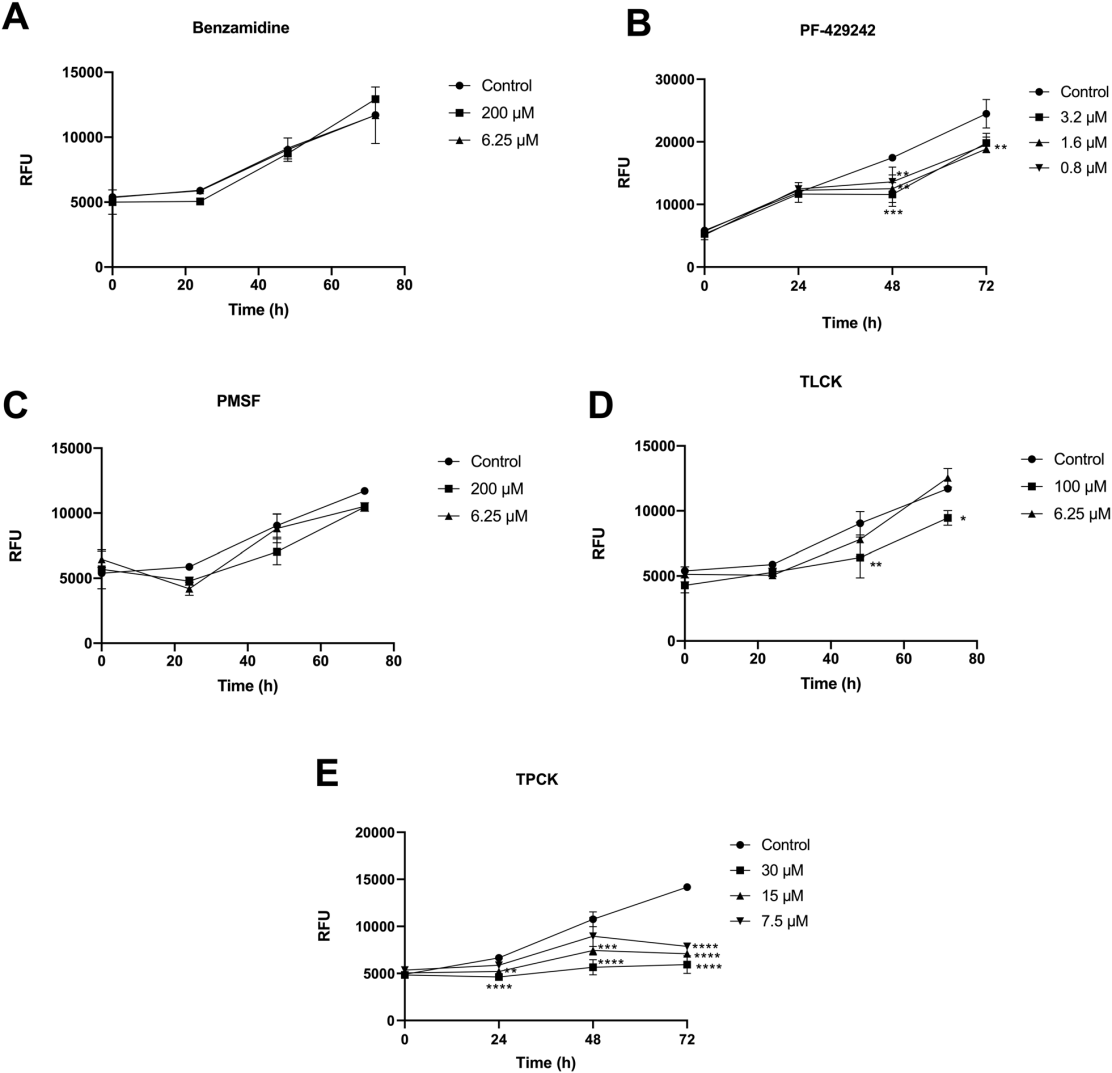
Effect of pretreatment with serine protease inhibitors on the infection of promastigotes in peritoneal macrophages. *L. amazonensis* promastigotes were incubated with the compounds for 1 h, washed with PBS and used to infect peritoneal macrophages. After the plates were scraped, the fluorescence intensity was determined at the following hours: 0 h, 24 h , 48 h, and 72 h. **A** Effect of treatment with benzamidine. **B** Effect of treatment with PF-429242. **C** Effect of treatment with PMSF. **D** Effect of treatment with TLCK. **E** Effect of treatment with TPCK. Graphs were constructed using the GraphPad Prism program. Statistical analyses were performed by a one-way ANOVA followed by Tukey’s post-test to compare the untreated control group with the other treatments: ****p < 0.0001, ***p < 0.001, **p < 0.01, *p < 0.05

Figure 2 shows that pretreatment with benzamidine and PMSF did not change the entry of promastigotes into macrophages and also did not alter the permanence and survival of amastigotes within these host cells (Fig. 2A, C, E). Regarding pretreatment with TLCK, the entry of promastigotes into the host cell occurred similarly to the control in both concentrations used (100 µM and 6.25 µM); however, after pretreatment with 100 µM, the survival of parasites after 48 h and 72 h of infection was slightly reduced (Fig. 2D). Pretreatment of promastigotes with PF-429242 and TPCK also did not alter the entry of promastigotes into macrophages, however, the survival of parasites throughout the first 72 h of infection was significantly altered (Fig. 2B, E). Parasites pretreated with PF-429242 had reduced survival, when compared with the control, 48 h and 72 h after infection, at the three concentrations used (3.2 µM, 1.6 µM, and 0.8 µM) (Fig. 2B). Parasites pretreated with TPCK had reduced survival, when compared with the negative control, 24 h (at 30 µM and 15 µM), 48 h (at 30 µM and 15 µM), and 72 h (at 30 µM, 15 µM, and 7.5 µM) after infection (Fig. 2E).

Supplementary Fig. 1 illustrates the effect of pretreatment with PF-429242 (3.2 µM) and TPCK (30 µM) on the survival of intracellular amastigotes of *L. amazonensis*. A reduction in the number of amastigotes, at 48 h and 72 h after infection, was observed in the groups in which promastigotes were pretreated before infection. It is interesting to highlight that no difficulty was observed in conversion of promastigotes into amastigotes, in parasites pretreated with PF-429242 or TPCK (Supplementary Fig. 1).

### Macrophage activation displays the same profile as nonactivated

**Fig. 3:**
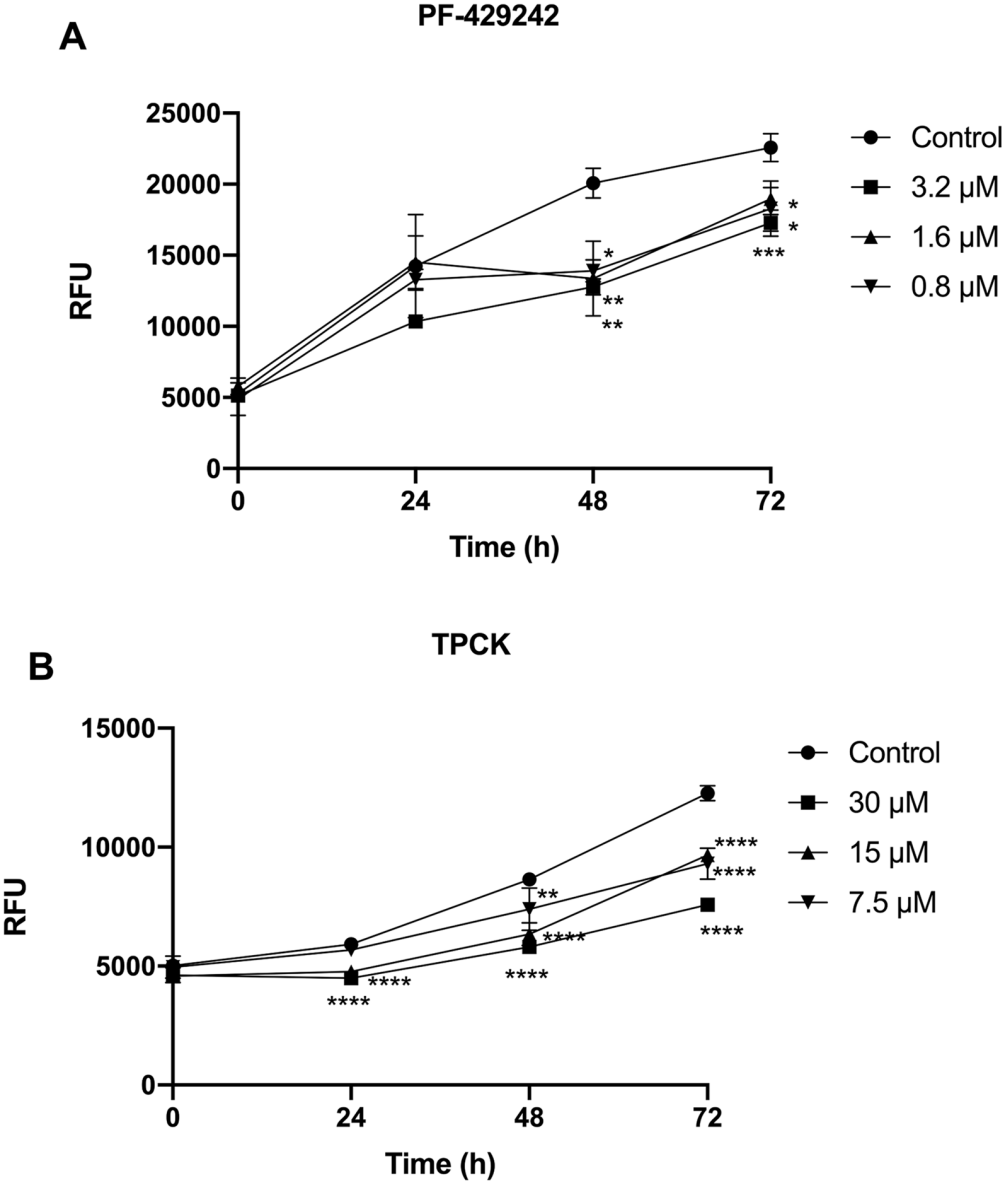
Effect of pretreatment with serine protease inhibitors on the infection of promastigotes in peritoneal macrophages prestimulated with IFN-γ. *L. amazonensis* promastigotes were incubated with the compounds for 1 h, washed with PBS, and used to infect peritoneal macrophages. Additionally, these macrophages were prestimulated with 0.5 ng/mL of IFN-γ overnight. After the plates were scraped, the fluorescence intensity was determined at the following hours: 0 h, 24 h, 48 h, and 72 h. **A** Effect of treatment with PF-429242. **B** Effect of treatment with TPCK. Graphs were constructed using the GraphPad Prism program. Statistical analyses were performed by a one-way ANOVA followed by Tukey’s post-test to compare the untreated control group with the other treatments: ****p < 0.0001, ***p < 0.001, **p < 0.01, *p < 0.05

As pretreatment with PF-429242 and TPCK caused greater changes in the intracellular survival of *L. amazonensis*, we used these compounds to pretreat promastigotes and to infect macrophages prestimulated with IFN-γ. Figure 3 shows that in macrophages prestimulated with IFN-γ, pretreatment with PF-429242 and TPCK also impairs the intracellular survival of *L. amazonensis*, as seen in non-prestimulated macrophages.

### The reduction in survival caused by pretreatment with PF-429242 and TPCK is not dependent on nitric oxide (NO) production

To evaluate whether the production of NO by host macrophages could be involved in the reduction in survival caused by pretreatment with PF-429242 and TPCK, we evaluated the production of NO in the supernatant of cultures of macrophages infected with pretreated promastigotes for 1 h with these compounds. We observed that there was no difference in NO production between the control group (macrophages infected with untreated promastigotes) and the groups that were infected with promastigotes pretreated with PF-429242 or TPCK (Supplementary Fig. 2), showing that it is not a mechanism dependent on NO production by host macrophages.

### PF-429242 and TPCK inhibited* L. amazonensis* serine protease activity

**Fig. 4:**
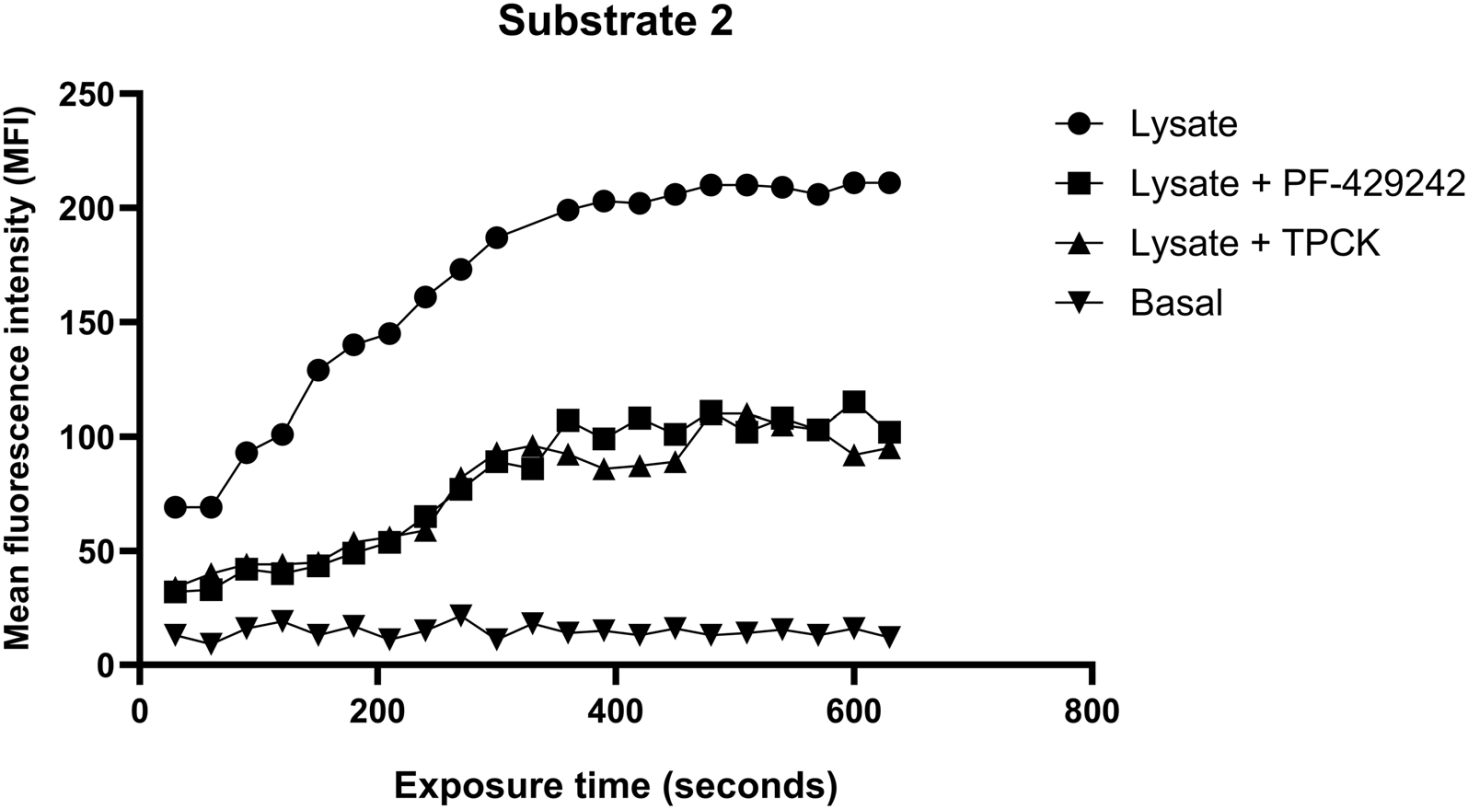
Enzymatic activity assay of *L. amazonensis* serine proteases. *L. amazonensis* promastigotes lysate was used in contact with a specific serine protease substrate (ABZ-V-F-R-S-L-K-Q EDDnp) and treated or not treated with 2.5 µM PF-429242 or TPCK. The reading was carried out using a spectrofluorimeter SoftMax M3 with excitation/emission of 320/420 nm. Subsequent readings were taken over the first 10 min

As PF-429242 and TPCK were the inhibitors that had the greatest impact on the survival of intracellular parasites, we evaluated their ability to inhibit the enzymatic activity of serine protease on *L. amazonensis*. For this, supernatant of *L. amazonensis* promastigotes lysate was used in contact with a specific serine protease substrate, base for subtilisin, without (negative control) or with 2.5 µM PF-429242 or TPCK. Figure 4 shows that both inhibitors were able to inhibit the enzymatic activity of *L. amazonensis* serine proteases. There was no impact on the activity when treated with E-64, an irreversible, potent and highly selective cysteine protease inhibitor, discarding participation of cysteine proteases in the cleavage of this substrate (Supplementary Fig. 3).

## Discussion

With no vaccines available for use in humans, chemotherapy for leishmaniasis constitutes one of the pillars for controlling the disease. However, the current chemotherapy available for the treatment of CL and VL presents many problems, which highlights the importance of new therapeutic alternatives [14]. An important strategy for discovering new antileishmanial agents is the discovery of cellular targets in *Leishmania* spp. Serine proteases have been highlighted as virulence factors in *Leishmania* spp. because they have important roles in the biological cycle of these parasites [9, 10]. *Leishmania* spp. have between 26 and 28 serine protease genes [15]. Different serine proteases have already been identified in *L. amazonensis*, such as oligopeptidase B (OPB) [16, 17] and subtilisin [10, 18]. Porta et al. [19] identified two targetable serine proteases in *L. mexicana* parasites, a carboxypeptidase LmxM.18.0450 and a prolyl oligopeptidase LmxM.36.6750 [19] A signal peptidase type I has also been identified in *L. major* [20].

Interestingly, serine protease inhibitors have shown effects on the viability and morphology of *L. amazonensis*. Souza-Silva et al. showed that an epoxy-α-lapachone inhibited *L. amazonensis* serine proteases, was effective against promastigotes and amastigotes of this *Leishmania* species, affected plasma membrane organization, and showed in vivo effect, causing reduction of the lesion size in mice infected with *L. amazonensis* [21]. Our research group has also demonstrated that protease inhibitors impair the viability of *L. amazonensis*. PF-429242, one of the key compounds used in this work, caused inhibition of the growth of promastigotes from different strains of *L. amazonensis* [18]. TPCK, another key compound used in this work, caused growth inhibition of promastigotes and intracellular amastigotes of *L. amazonensis* [22]. Additionally, in *L. amazonensis* promastigote forms, TPCK induced mitochondrial changes, caused oxidative stress, and altered the lipid content, while in amastigotes, it induced the appearance of cytoplasmic vacuoles. In the same work, it was shown that TPCK also had an effect in vivo, causing a reduction in lesion size and parasite load in mice infected with *L. amazonensis* [22].

In the present work, we expanded the tests with protease inhibitors, also testing benzamidine, PMSF, and TLCK, in addition to PF-429242 and TPCK, already shown previously [18, 22, 23]. Benzamidine and PMSF had no effect on *L. amazonensis* promastigotes and amastigotes, while TLCK was effective on both evolutionary forms. However, TLCK did not show selectivity for the parasite, as it was slightly toxic to host cells. From this, it can be concluded that the recently tested inhibitors did not show an improvement in leishmanicidal activity when compared with PF-429242 and TPCK.

In work by Silva-Lopez et al. [24], 1 mM benzamidine reduced the viability of *L. amazonensis* promastigotes after 24 h, 48 h, and 72 h of treatment, 100 µM TLCK did not reduce promastigote viability, while TPCK was the most effective, reducing cell viability at 100 µM after 4 h, 24 h, 48 h, and 72 h of incubation. [24]. These results are partially similar to ours, in which we showed that TPCK was one of the best inhibitors, together with PF-429242. However, in the present study, TLCK showed discrete activity in *L. amazonensis* promastigotes, with an IC_50_ value of 52.54 µM. Additionally, Chakraborti et al., [25] tested the inhibitors aprotinin, benzamidine, TLCK, and TPCK and showed that aprotinin (1 µM) and benzamidine (1 mM) were the ones that best inhibited the activity of *L. donovani* serine proteases, inhibiting 100% and 72% of the proteolytic activity, respectively. TPCK and TLCK inhibited 52% and 18% of the proteolytic activity, respectively. Furthermore, aprotinin (1 µM) and benzamidine (1 mM) reduced the cell viability of *L. donovani* promastigotes after 5 h, 10 h, 15 h, and 20 h of incubation, while TPCK and TLCK only did so after 15 h and 20 h, unlike the present study, in which TPCK proved to be one of the most efficient inhibitors [25]. However, it is interesting to highlight that the concentration of benzamidine used in the studies cited in this paragraph was much higher than that used by us.

To investigate the role of protease inhibitors in the infection of peritoneal macrophages by *L. amazonensis*, we pretreated promastigotes with different concentrations of the inhibitors during 1 h and after washing with PBS, they were placed in contact with peritoneal macrophages. Pretreatment with benzamidine and PMSF did not change the entry of promastigotes into macrophages and the survival of amastigotes within these host cells. After pretreatment with TLCK, the entry of promastigotes into the host cell also occurred similarly to the control; however, after pretreatment with 100 µM, the survival of parasites after 48 h and 72 h of infection was slightly reduced. Pretreatment of promastigotes with PF-429242 and TPCK also did not alter the entry of promastigotes into macrophages; however, the survival of parasites throughout the first 72 h of infection was significantly altered. This also occurred in macrophages pretreated with IFN-γ, showing that the activation of host cells did not interfere with the kinetics of reduced survival caused by pretreatment with PF-429242 and TPCK. Chakraborti et al. [25] applied a pretreatment of *L. donovani* promastigotes with different inhibitors for 2 h, followed by infection of RAW 264.7 cells and showed that aprotinin and benzamidine were the inhibitors that most reduced the intracellular survival of the parasites, followed by TPCK and TLCK. Nevertheless, in the present study, pretreatment of *L. amazonensis* promastigotes for 1 h, before infection of peritoneal macrophages, showed that TPCK and TLCK had a greater implication in reducing the intracellular survival of parasites, when compared with benzamidine. However, it is worth highlighting that the concentration of benzamidine used in the present study was five times lower [25].

Interestingly, the reduction in intracellular survival caused by pretreatment with these compounds is not dependent on NO production by macrophages. These results show that the treatment of promastigotes with different protease inhibitors alters the survival of the parasite within macrophages. This can be explained by the inhibition of serine proteases caused by these compounds. As also shown in this study, PF-429242 and TPCK are capable of inhibiting the enzymatic activity of *L. amazonensis* serine proteases.

It is important to highlight that promastigotes treated for 1 h with inhibitors, at the same concentrations of infection in the macrophages experiment, did not change their survival when replaced in culture medium supplemented with 10% FBS, after 4 h, 24 h, 48 h, and 72 h of incubation. This fact shows that the reduction in parasite survival within macrophages after pretreatment with PF-429242, TLCK, and TPCK did not occur owing to their death during 1 h of treatment. This short treatment interval caused changes in the promastigotes, one of which may be the inhibition of serine proteases, which resulted in the subsequent reduction of the parasites’ survival within the macrophages. This fact shows that *Leishmania* parasites need their serine proteases to survive inside macrophages, which may be necessary for the uptake of nutrients within the parasitophorous vacuole, act as growth factors for intracellular amastigotes, or to participate in the parasite defense against defensins or other antimicrobial peptides.

Using transgenic parasites for serine proteases has demonstrated different outcomes. Parasites *L. major* null mutant of signal peptidase type I was not possible to create, suggesting that these proteases are essential for parasite survival. The heterozygote mutant was obtained by disrupting one allele of signal peptidase type I in *L. major*, and it showed significantly reduced level of infectivity in bone marrow-derived macrophages and was enable to cause cutaneous lesions in BALB/c mice [11]. In addition, *L. major* parasites with knockout for oligopeptidase B were significantly less able to infect and survive within macrophages in vitro [26]. *L. donovani* knockout for subtilisin showed reduced virulence in vivo models and had difficulty converting promastigotes into amastigotes [10]. In our study, the use of serine protease inhibitors did not alter the entry of promastigotes inside macrophages and did not alter the differentiation for amastigotes, but it did affect the survival inside macrophages, indicating a different phenotype using this not lethal strategy. As we demonstrated the capacity in the inhibition of serine protease activity by PF-429242 and TPCK, in *L. amazonensis*, using the nonlethal pretreatment, subtilisin probably did not participate in amastigote differentiation but in the intracellular survival.

## Conclusion

Our results highlight the leishmanicidal potential of the serine protease inhibitors, especially PF-429242 and TPCK, and show that the inhibition of serine proteases by different serine protease inhibitors can compromise the survival of these parasites inside host macrophages without altering the entry of promastigotes into these cells, highlighting the importance of serine proteases for *L. amazonensis*, as well as for other *Leishmania* species.

## Supplementary Information

Supplementary material 1.

## Data Availability

No datasets were generated or analyzed during the current study.
