# Supplementary material for: Pretreatment with serine protease inhibitors impairs Leishmania amazonensis survival on macrophages
Source: Parasit Vectors. 2025 Jan 23;18:23. doi: 10.1186/s13071-024-06630-w (PMC11760092; doi:10.1186/s13071-024-06630-w)
Supplement: Supplementary file 1 — Supplementary material 1. [file 13071_2024_6630_MOESM1_ESM.pdf]

Supplementary Material

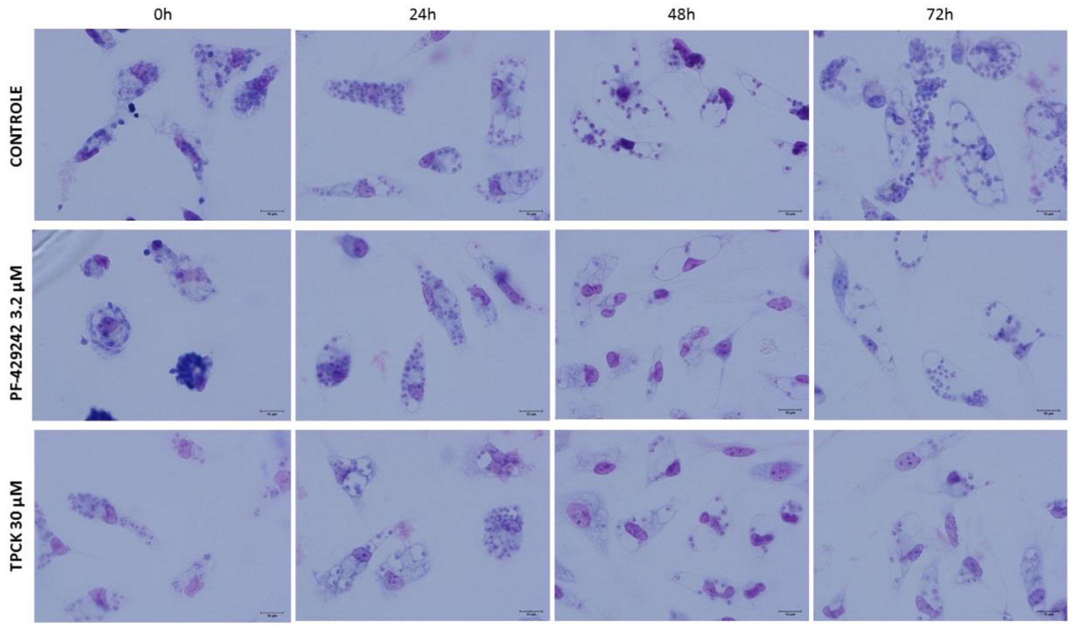

Supplementary Figure 1: Illustrative photomicrograph of the effect of pre-treatment of *L. amazonensis* promastigotes with protease inhibitors on the infection of peritoneal macrophages. *L. amazonensis* promastigotes were incubated with the PF-429242 or TPCK for 1 h, washed with PBS e and used to infect peritoneal macrophages for 4h. After coverslips were removed at times 0, 24, 48 and 72h, fixed with etanol, stained with Giemsa and photographed.

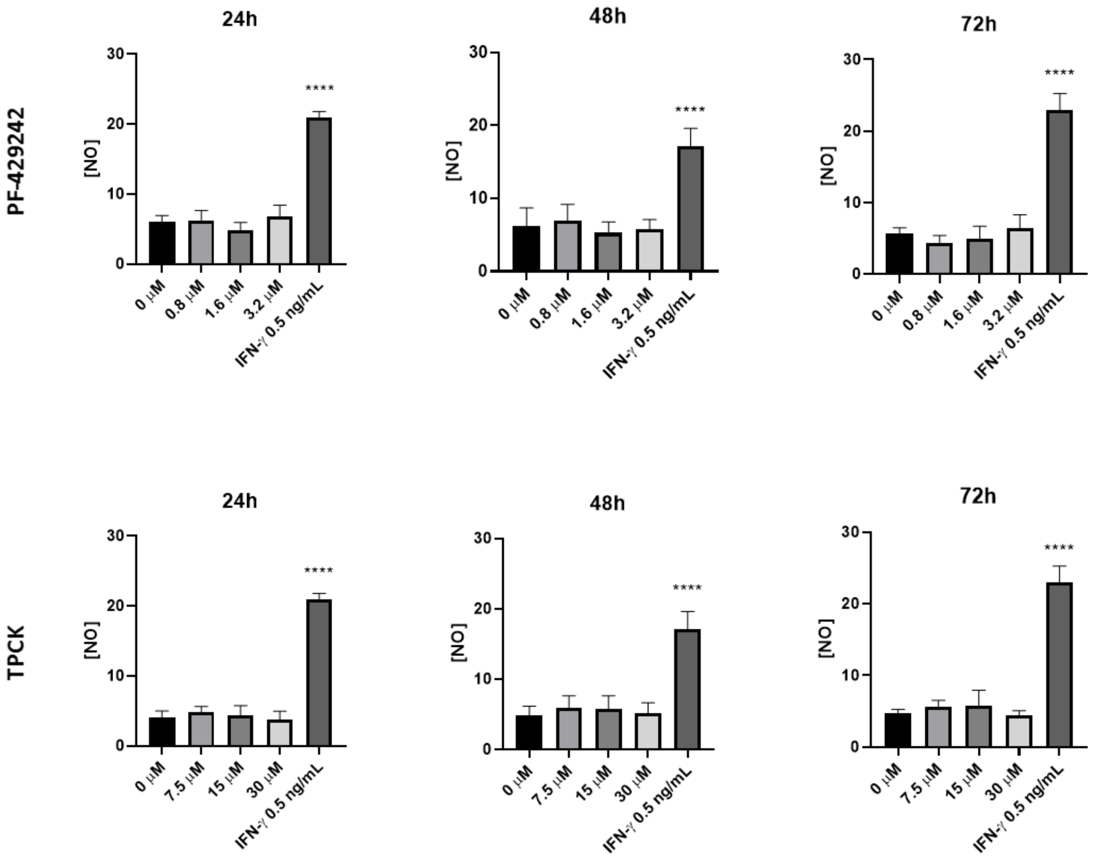

Supplementary Figure 2: Nitric oxide (NO) levels from cultures of *L. amazonensis*-infected macrophages. Supernatant from cultures of *L. amazonensis*-infected macrophages, in which promastigotes were pretreated or not with with PF-429242 (3.2, 1.6 and 0.8 µM) or TPCK (30, 15 and 7.5 µM), was collected after 24, 48 and 72h after infection and NO levels were determined by the Griess method. Macrophages pre-stimulated with 0.5 ng/mL IFN-ɣ overnight and infected with *L. amazonensis* promastigotes without pretreatment were used as positive control. Graphs were constructed using the GraphPad Prism software. Statistical analyses were performed by a one-way ANOVA followed by Dunnett's post-test to compare the untreated control group with the other treatments: **** $p < 0.0001$.

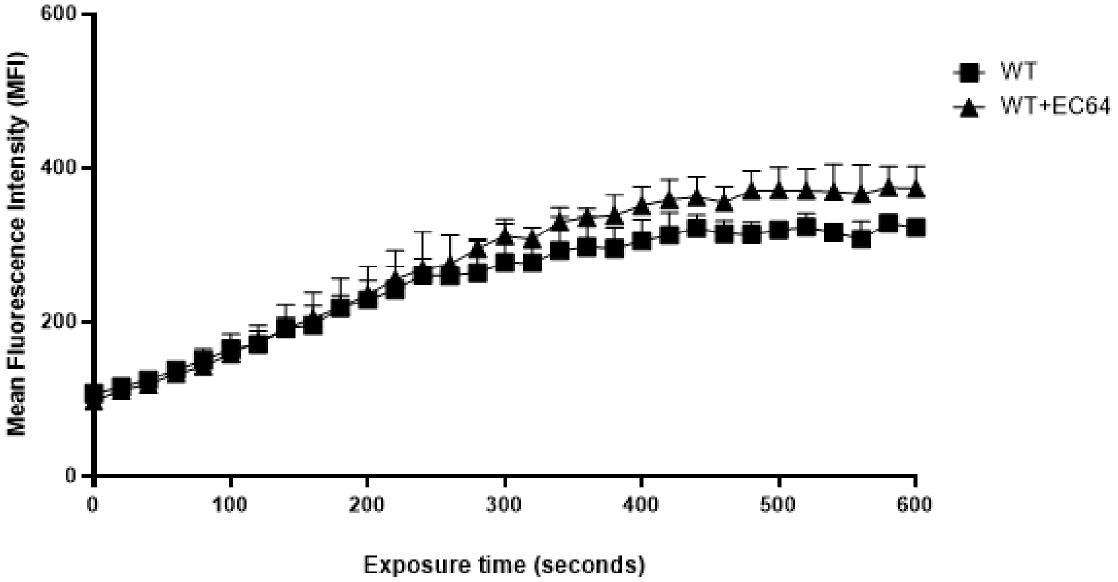

Supplementary Figure 3: Enzymatic activity assay of *L. amazonensis* using cysteine protease inhibitor. Supernatant from *L. amazonensis* promastigotes lysate was exposed to a specific serine protease substrate (ABZ-V-F-R-S-L-K-Q EDDnp) incubated with 3µm of E-64 (cysteine protease inhibitor). Subsequent measurements were taken during the initial 10 minutes. The measurements were conducted using a SoftMax M3 spectrofluorimeter with excitation/emission wavelengths of 320/420 nm.
